# Effects of Cations on HPTS Fluorescence and Quantification of Free Gadolinium Ions in Solution; Assessment of Intracellular Release of Gd^3+^ from Gd-Based MRI Contrast Agents

**DOI:** 10.3390/molecules27082490

**Published:** 2022-04-12

**Authors:** Angelo Scarciglia, Enza Di Gregorio, Silvio Aime, Giuseppe Ferrauto

**Affiliations:** Molecular Imaging Center, Department of Molecular Biotechnologies and Health Sciences, University of Torino (IT), Via Nizza 52, 10126 Turin, Italy; angelo.scarciglia@edu.unito.it (A.S.); silvio.aime@unito.it (S.A.); giuseppe.ferrauto@unito.it (G.F.)

**Keywords:** calcium, cations, fluorescence, gadolinium, HPTS, pH, pyranine, lanthanides, probe, quantification, release

## Abstract

8-Hydroxypyrene-1,3,6-trisulfonate (HPTS) is a small, hydrophilic fluorescent molecule. Since the pKa of the hydroxyl group is close to neutrality and quickly responds to pH changes, it is widely used as a pH-reporter in cell biology for measurements of intracellular pH. HPTS fluorescence (both excitation and emission spectra) at variable pH was measured in pure water in the presence of NaCl solution or in the presence of different buffers (PBS or hepes in the presence or not of NaCl) and in a solution containing BSA. pKa values have been obtained from the sigmoidal curves. Herein, we investigated the effect of mono-, di-, and trivalent cations (Na^+^, Ca^2+^, La^3+^, Gd^3+^) on fluorescence changes and proposed its use for the quantification of trivalent cations (e.g., gadolinium ions) present in solution as acqua-ions. Starting from the linear regression, the LoD value of 6.32 µM for the Gd^3+^ detection was calculated. The effects on the emission were also analyzed in the presence of a combination of Gd^3+^ at two different concentrations and the previously indicated mono and di-valent ions. The study demonstrated the feasibility of a qualitative method to investigate the intracellular Gd^3+^ release upon the administration of Gd-based contrast agents in murine macrophages.

## 1. Introduction

The use of fluorescent dyes in life science is very wide. They have been employed for many applications in preclinical research (e.g., fluorescent cells microscopy, in vivo imaging, protein structure analysis, photodynamic therapy, etc.) but also as in vitro biosensors for the detection and quantification of parameters of interest in the diagnosis and treatment of diseases [1,2,3,4,5].

Different classes of fluorescent probes have been developed as (i) inorganic pigments [6], (ii) organic dyes (e.g., fluorescein, cyanines, etc.), (iii) fluorescent proteins (GFP, RFP, etc.), (iv) inorganic nanoparticles (e.g., quantum dots, etc.), (v) fluorescent-nanosystems (e.g., liposomes or mesoporous silica nanoparticles filled with fluorescent molecules) [7], (vi) lanthanide fluorescent complexes [8,9].

An important biomedical application of fluorescent dyes relies on their use as responsive agents, which are able to provide in vitro and/or in vivo insights into bio-chemical events occurring in cells systems. The most common way to use such responsive agents is represented by the possibility to develop ratiometric approaches to quantify the parameter of interest, independently from knowing the actual probe concentration but simply analyzing excitation and/or emission spectra at different wavelengths [10,11]. In this way, several bio-chemical parameters can be quantified as pH [12], enzyme activity [13,14], concentration of reactive oxygen species [15,16], glucose [16,17], H_2_O_2_, ions [18,19], drugs [20], soluble amyloid protein [21], etc.

A widely exploited example of fluorescent responsive probe is represented by organic dye able to change their fluorescence on the basis of the *bulk* pH. (i.e., fluorescent pH-probes) [22]. The quantitative assessment of the pH has been demonstrated to be important for the study of cell biology (e.g., intracellular trafficking of organelles [23], analysis of membrane transporters, etc.).

**Figure 1:**
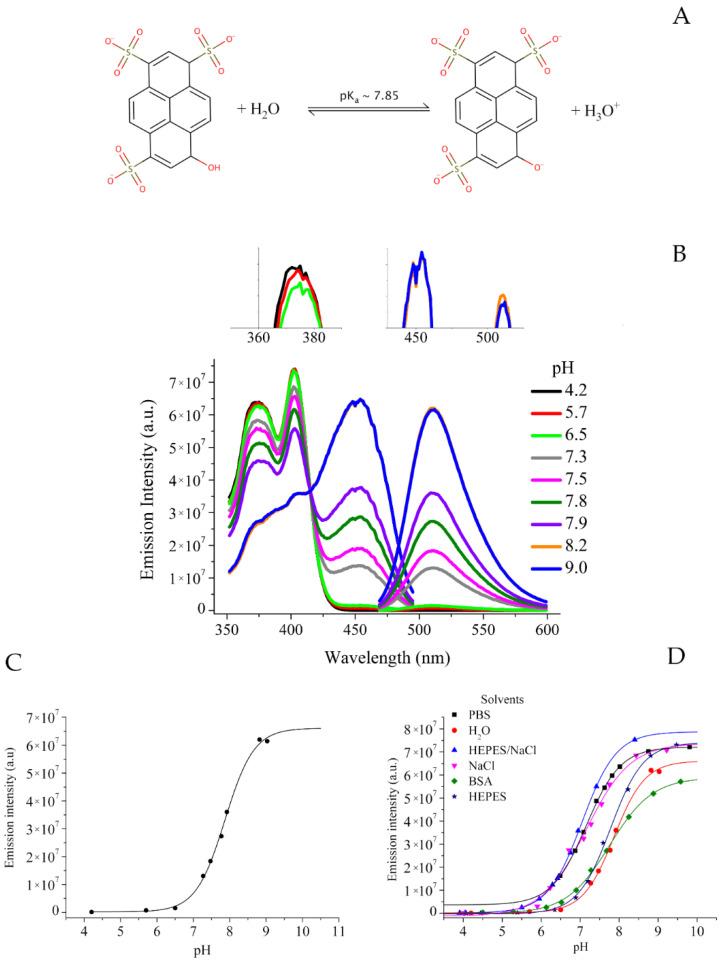
(**A**) Equilibrium reaction with calculated pK_a_ value. (**B**) Excitation and emission spectra in water at variable pH values. (**C**) Sigmoidal behavior of the emission intensity at variable pH in water. (**D**) Sigmoidal behavior of the emission intensity at variable pH in different media.

One of the most interesting fluorescent molecules for biological applications is represented by 8-hydroxypyrene-1,3,6-trisulfonate (pyranine or HPTS, *Chemical structure* in Figure 1A). This dye has been shown to be very interesting for many applications since it displays a series of features making feasible its application in biomedicine. First of all, HPTS is a small water soluble molecule (M.W. is 524.39 g/mol). It is a highly photostable, bright yellow dye with an intense, vibrant fluorescence. It has a generally low toxicity and it does not interfere with biological pathways, so it can be applied for cells experiments. The most interesting feature of HPTS is the possibility to establish a fluorescence-based ratiometric method to quantify medium pH, without the need of knowing the actual probe concentration. Typically, ratiometric pH detection requires the combination of one pH-sensitive and one pH-insensitive fluorescent dye. On the contrary, HPTS allows ratiometric detection of pH from the comparison of emission intensity (λ = 511 nm) upon excitation at two different wavelengths (λ = 414 and 450 nm).

Another important advantage of HPTS is that pK_a_ of the phenol group (the group responsive to pH) is near the neutrality (ca. 7.3, depending on the medium), making this probe responsive to pH variation in the 5–9 pH range and well suitable for biomedical applications. Furthermore, changes in fluoresce respond rapidly to pH changes.

Finally, HPTS is a membrane impermeant probe because of the highly negative charge provided by sulfonic groups, which are generally deprotonated at all pH values compatible with biological systems [24].

Thanks to all these features, HPTS has largely been employed to measure intracellular pH but also for other applications in biomedicine as the study of protein structure and modifications, as metal ion-sensing, and as pH sensors for the monitoring of chronic wound pads [25,26,27].

However, the use of HPTS is not limited to the simple native molecule, but a series of applications have been reported where HPTS has been chemically modified to change pK_a_ properties or to produce glucose biosensors [28,29] or chemically bound to polymers and/or other chemical structures for generating innovative pH biosensors [30,31,32,33]. More recently, supramolecular host-guest adducts of HPTS have been studied as ways to improve cell internalization or in the context of exploiting hydrophobic interactions with MRI contrast agents [34].

Even if the application of HPTS is widely diffuse and many studies on this molecule have been reported, some important issues remains unclear and not completely understood. One of these relies on the study of interference on the fluorescence triggered by the presence of anions and cations in the environment. *Avnir Y.* and *Barenholz Y*. started paying attention to possible medium-related artifacts on pH determination by pyranine [35].

The herein reported study aims at analyzing the effect of mono-, di- and tri-valent cations on HPTS fluorescence and the changes in HPTS fluorescence at the different buffers normally employed in cells’ studies. Primary attention has been devoted to the fluorescence response in the presence of Gd^3+^ in the free form. The sensibility to the concentration of the free cations in solution makes it possible to determine their presence quantitatively but also to use it as an intracellular qualitative probe able to determine the *in cell* release of the free Gd(III) ions after the administration of a Gadolinium-based contrast agent (GBCA) for Magnetic Resonance Imaging (MRI) [36,37,38,39,40,41]. This issue is of primary importance for the chemistry of MRI contrast agents, since it has previously established that the stability of such agents can be strongly hampered when intracellularly entrapped [42] and that in vivo, the release of Gd(III) is correlated to a high toxicity [43,44] and to the eventual occurrence of Nefrogenic Systemic Fibrosis (NSF) [45,46] and Gd-retention and tissue accumulation [45].

## 2. Results

### 2.1. Effect of Media on HPTS Fluorescence

The fluorescence of HPTS at different pH values (generally from 4.0 to 9.0) has been tested in the presence of different media.

In particular, HPTS has been dissolved in pure water, in NaCl solution, in Hepes buffer, Hepes/NaCl buffer, in PBS (phosphate buffer saline) and in BSA (bovine serum albumin) containing NaCl solution.

HPTS is present in two chemical forms depending on pH, i.e., with protonated and de-protonated hydroxyl group (Figure 1A, HPTS-OH vs. HPTS-O^-^).

Figure 1B shows excitation and emission spectra of HPTS in water at variable pH values. In particular, excitation spectra have been acquired in the 350–500 nm range by fixing λ^em^ = 511 nm.

As it is visible, two peaks are present in the 350–420 nm range whose intensity increases by lowering pH (the peak at 403 nm is maximum at acidic pH). This signal derives from the presence of the protonated hydroxyl group of HPTS.

On the contrary, the intensity of the peak at 450 nm increases directly proportional to pH increase (maximum at alkaline pH values). This signal derives from the presence of the de-protonated hydroxyl group of HPTS. The isosbestic point is at 413 nm.

Emission spectra have been acquired in the 470–600 nm range by fixing λ^exc^ = 450 nm. The fluorescence emission intensity (λ = 511 nm) depends on the pH. The intensity of fluorescence emission has been plotted against pH values by obtaining the sigmoidal curve reported in Figure 1C. From the fitting of this curve, the pKa value of the OH group can be extracted (Table 1).

It is to be noted that the used concentration of HPTS has been preliminarily chosen to avoid the quenching effect, on the basis of results obtained by acquiring spectra at variable concentration. Data have been reported as Figures S1 and S2 and Table S2. As the tested experimental conditions revealed, at high HPTS concentration, the linearity was lost, therefore, the reference HPTS is set at 10 µM in order to maintain the linear range in the emission intensities.

The same experiments have been carried out by using the different media. Sigmoidal curves have been compared in Figure 1D (all the parameters of the sigmoidal fitting are available in Table S1).

It is possible to note that the curve moves to the left when passing from pure water to NaCl containing media, so reporting on the effect of sodium chloride salt on pH quantification by HPTS fluorescence. No significant differences have been gained by moving from NaCl solution to Hepes/NaCl or PBS/NaCl. Whenever NaCl concentration is fixed, the same sigmoidal curve is present.

In analogy, no significant difference is present by moving from pure water to Hepes or PBS buffer (both w/o NaCl). These data report on the effect of NaCl.

In the case of BSA, the effect is reasonably due to the negative charge of BSA at the tested pH values (the BSA isoelectric point is 4.9). This can sequester Na^+^ cations, so reducing the enhancement of the expected HPTS emission intensity. As consequence, a shift of the sigmoid is present [47].

All together, these data indicate the need for caution when HPTS is used for measurement of pH, especially in intracellular compartments, since it is necessary to know the exact composition of the microenvironment in which the fluorescence probe distributes.

### 2.2. Effect of Cations on HPTS Fluorescence

Starting from the above reported data, the effect of cations has been investigated more in detail. Cations can interact with negatively charged sulfonic groups and with the deprotonated HPTS-O^-^ form by stabilizing it. In this way, in the presence of the cations, there is an enhancement of the intensity of the peak at 450 nm (quite analogous to what happens at alkaline pH values).

Na^+^, Ca^2+^, and La^3^^+^ chloride salts have been tested as representative of mono-, di-, and tri-valent cations. The fluorescence of HPTS water solutions at fixed pH = 6.0 (to avoid the precipitation of hydroxides of cations) has been tested at variable concentration of cations in the 0.05–0.3 mM range.

**Figure 2:**
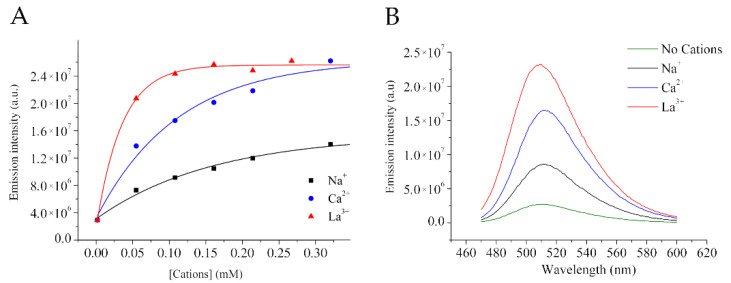
(**A**) Emission intensity increases by increasing cations concentration. (**B**) Comparison of emission spectra for 0.1 mM of cations and 10 μM HPTS. (pH = 6.2 ± 0.1).

As reported in Figure 2A,B, by increasing cations concentration there is an increase in the intensity of fluorescence at emission at 511 nm upon excitation 450 nm. The curves reach a plateau value. The effect is in the order monovalent > divalent > trivalent.

As it is possible to extract from the curves, when 10 µM HPTS are placed in the presence of 0.1 mM of cations, there is an enhancement of ca. 200% (for Na^+^), ca. 500% (for Ca^2+^), and ca. 730% (for La^3+^) (Figure 2A). The parameters of the exponential fittings represented in Figure 2A are available as Table S3.

Reported data (Figure 2 and Figure S3) are well in line with what previously reported by Avnir Y. et al. who analyzed the effect of different salts on pH determination by HPTS [35]. They noted that inaccuracy of pH determination by HPTS depends on cations and anions present in solution based on their order in the Hofmeister series.

Herein, we confirm this trend because the enhancement of HPTS fluorescence is K^+^ <Na^+^ < Mg^2+^< Ca^2+^< La^3+^ (in line with Hofmeister series).

### 2.3. Use of HPTS to Determine Free Gd^3+^ in Solution

Starting from the above reported considerations, HPTS has been herein proposed as an innovative tool for the quantification of free lanthanide ions present in solution with a special focus on gadolinium. This aim is important for many applications as: (i) the chemical synthesis of MRI Gd-based contrast agents (where the concentration of free Gd^3+^ must lower than 0.3% mol/mol of total complex in solution), (ii) the quantification of free Gd^3+^ in the environments (as pollutant), and (iii) the quantification of free Gd^3+^ in biological systems (because of gadolinium toxicity).

Gd(III) chelates are often used as contrast agents (CAs) for MRI applications. Their use relies on the high thermodynamic and kinetic stability, which hampers the release of free Gd^3+^ ions, which are highly toxic for living systems [43]. Generally, the chemical synthesis of Gd complexes requires caution in order to prevent the presence of free metal ions and to ensure the completion of the complexation step [48]. It is widely accepted that a Gd complex must have less 0.3% mol:mol of free Gd^3+^ ions. Hence, a method to quantify the free Gd^3+^ ions in the specimens is needed [48].

**Figure 3:**
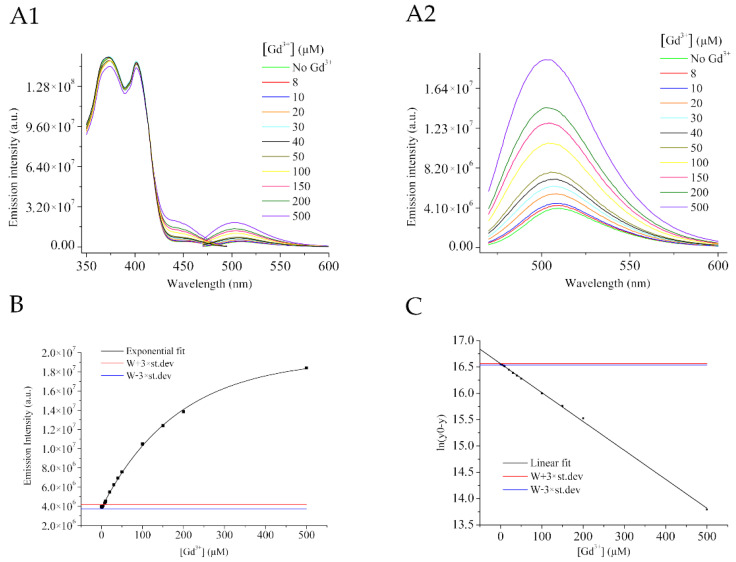
(**A1**) Excitation and emission spectra at different Gd^3+^ concentrations. (**A2**) Magnification of the emission spectra. (**B**) Emission intensity at different Gd^3+^ concentrations. (**C**) Linearized fitting of data reported in (**B**). (pH = 6.1 ± 0.1).

To define a reliable method for the quantification of the trivalent ions in solution, emission and excitation spectra of HPTS fluorescence at different concentrations of Gd^3+^ in the range 8–500 µM have been acquired (Figure 3A,B).

It is of immediate interpretation how after increasing the Gd^3+^ concentrations, also the relative emission intensity increases in agreement with what was previously demonstrated by the other lanthanide La^3+^.

Plotting the measured emission intensities at the different Gd^3+^ concentrations it is possible to notice an exponential behavior in the distribution of the points. As a consequence, an exponential fitting analysis was conducted, revealing a good R^2^ value, with obtained parameters summarized in Table S4.

The exponential curve has been linearized (Figure 3C) as reported in Materials & Methods.

Table S5 summarizes summarizing the linear fitting parameters.

When 10 µM of HPTS in water is employed, the calculated Limit of Detection (LoD) for Gd^3+^ corresponds to 6.32 µM.

ln(y0 − y) = ln(1.94 × 10^−7^ − 4.3 × 10^6^) => X = 6.32 µM (1)

The expLoD, as the minimum concentration experimentally determined, is 8 µM.

### 2.4. Matrix Effect

Since the interactions of HPTS are not specific for lanthanides, but they occur also with mono- and divalent cations, the effect of metal interference on the determination of [Gd^3+^] has been tested. A matrix effect on HPTS spectra induced by the mono- or divalent cations, in the presence of a combination of HPTS and Gd^3+^, was considered with the lantanoid atom tested at different concentrations.

**Figure 4:**
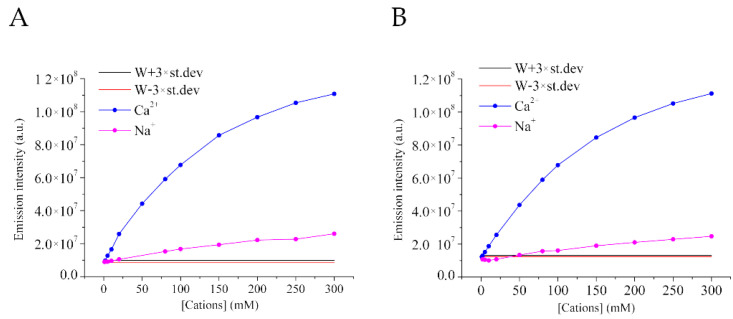
(**A**) Emission intensities at (Gd^3+^) = 90 µM. (**B**) Emission intensities at (Gd^3+^) = 45 µM. (pH = 6.1 ± 0.1).

The first step was the evaluation of how the emission intensity changed adding an increasing amount of mono- or divalent cations (Na^+^ and Ca^2+^, respectively) to a standard solution containing HPTS 10 µM and Gd^3+^ 45 µM. The concentration range considered for the added cations was 1–300 mM. Fixing the trivalent cation, the presence of the divalent one has a more intense effect on enhancing the HPTS emission with respect to the monovalent one, with an emission out of the blank range, further strengthening the hypothesis previously reported (Section 2.2) (Figure 4B).

Then, the idea was to evaluate if increasing Gd^3+^ concentration maintaining the same amount of HPTS in tested solutions, as of Na^+^ and Ca^2+^, the effect persists (Figure 4A). Increasing the fixed Gd^3+^ concentration up to 90 µM, a similar behavior as the one described before is maintained.

**Figure 5:**
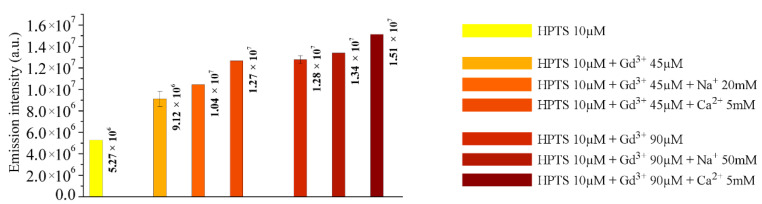
Column plot comparing the extrapolated emission intensities of the samples. This defines for each experimental condition the minimum concentration of mono- and divalent cations able to induce the matrix effect.

Figure 5 reports which are the minimum concentrations of the di- and trivalent ions, for the fixed Gd^3+^ concentrations, able to determine a significant effect on the emission intensities.

These values will define the detection limits in the described experimental conditions, which are attributable to the presence of a matrix effect. In the standard solution containing only Gd^3+^ and HPTS, as above reported, there is an increase in the emission intensity by increasing the Gadolinium content.

### 2.5. Use of the HPTS as an Intracellular Probe

**Figure 6:**
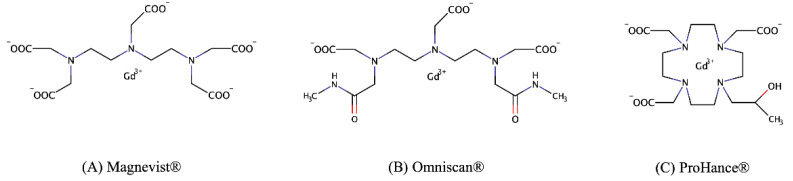
(**A**) Magnevist^®^ structure, (**B**) Omniscan^®^ structure and (**C**) ProHance^®^ structure.

The ability of HPTS fluorescent dye to sense the presence of free Gd^3+^ in the intracellular compartment has been tested. As *proof-of-concept,* J774A.1 murine macrophages have been co-incubated in the presence of HPTS (as the fluorescent reporter) and the well-known Omniscan^®^, Magnevist^®^, and ProHance^®^ MRI contrast agents (Figure 6).

Their use has been considered due to their different stability, which is evaluated as their capacity to release Gd^3+^ in the unchelated form [42]. At five hours post-incubation of the J774A.1 cell line, the amount of Gadolinium released by Omniscan^®^ should be near 40% of the loaded amount, whereas the other agents can be found almost totally as an intact complex at the same time point [42]. Replicates for each of the five experimental conditions of incubation (only medium, HPTS alone, and HPTS plus the three different Gd-based contrast agents) help demonstrate the qualitative power of the method in determining the presence of intracellular released Gd^3+^.

**Figure 7:**
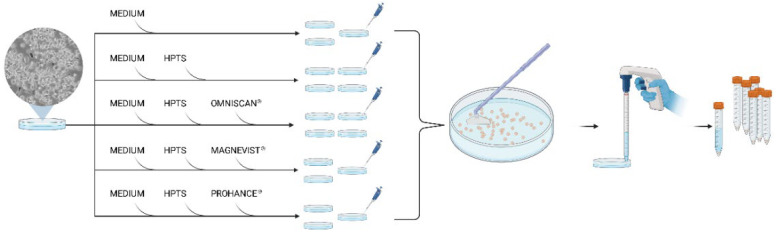
Graphical representation of experimental setup, highlighting the use of replicates for statistical analysis. The incubation conditions have been reported, followed by detachment of the cells by the use of a scraper and washes with fresh PBS. Samples finally transferred into falcon for the subsequent pre-lysis fluorescence acquisition.

After the incubation, cells have been extensively washed with fresh PBS buffer to eliminate unloaded Gd complexes and HPTS. Then, the cells have been detached and suspended in PBS buffer for the acquisition of the emission fluorescence spectra (Figure 7).

**Figure 8:**
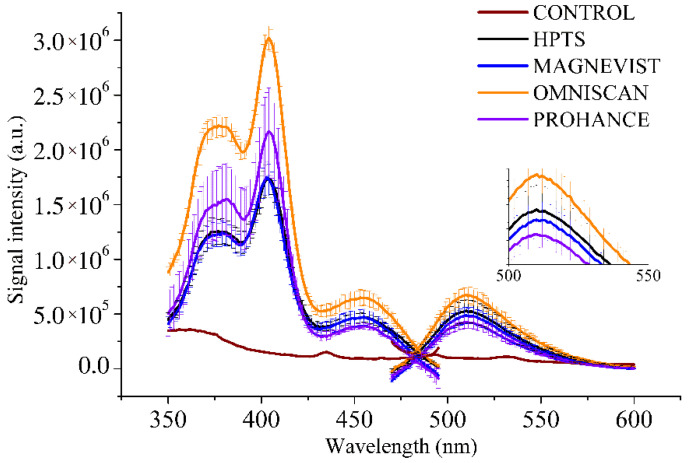
Excitation and emission spectra acquired before cell lysis.

As expected, the signal intensity arising from intact cells reflects the stability properties of the MRI agents (Figure 8). The spectra with the highest intensity arise from the cell samples incubated with HPTS plus Omniscan^®^ (Figure 8, orange lines). They are significantly different from the spectra of the cells incubated with Magnevist^®^ or ProHance^®^ (still in the presence of the same amount of HPTS) or HPTS alone which are found to be aligned in intensity.

Spectra have been acquired also after the lysis of the samples. This provided to the fluorescent system an increase in the cations (Na^+^) available for the interactions with HPTS, due to the presence of PBS in the extracellular space, with a concentration one order of magnitude higher (140 mM) than the physiological one for the intracellular compartment (12–14 mM). As previously demonstrated, the presence of such a high Na^+^ concentration can significantly affect the HPTS fluorescence, hiding the effect of the Gd^3+^, by changing the spectra profiles as reported in Figure S5. The PBS-related masking effect can be excluded in the pre-lysis condition where the competitive Na^+^ is only provided by the intracellular compartment.

To further validate the hypothesis, it appeared necessary a normalization of all the data, as a function of the cell number, quantified by the use of the Bradford assay. We calculated the amount of fluorescent dye in each cell. A calibration curve set on a variable HPTS concentration, in PBS (Figure S4, Table S6), allowed the quantification of the number of HPTS molecule *per* cell, which comes out to be all in the same order of magnitude (3–4 × 10^7^ molecules/cell, Table S7).

The total amount of Gadolinium both chelated and in the free form, present in each sample, has been calculated using a *T*_1_ relaxometric approach, after sample mineralization [49]. Even if a slightly higher concentration of the lanthanide per cell was present in the sample containing ProHance^®^ (5.3 × 10^10^ Gd^3+^/cell), the maximum effect in terms of enhancing the signal intensity in the profiles comes from a sample containing a lower total concentration of Gadolinium, which is the one containing Omniscan^®^ (4.4 × 10^10^ Gd^3+^/cell). 

It should be noted that in the case of ProHance^®^ an increase in the fluorescence signal might also arise from the intact complex as the result of the formation of a reversible adduct between HPTS and ProHance^®^. In spite of this additional contribution and the slightly higher amount of Gd ions, the qualitative test showed that the release of Gd from Omniscan^®^ causes a definitively higher response.

All this demonstrates that the effect on the spectra is due to the amount of free trivalent ion available, released by the contrast agent, maximum when incubated with Omniscan^®^ (Table S8).

## 3. Discussion

HPTS is widely exploited as a pH indicator due to the possibility to observe an altered emission profile at variable pH values and for the high biocompatibility [24]. In this study, we demonstrated how excitation and emission spectra are able to sense not only a variable H^+^ concentration but also the presence of mono-, di- and trivalent cations. Therefore, the HPTS fluorescence at variable pH was assessed on different media, so reporting on the influence of media on the revealed fluorescence.

In particular, we demonstrated how the presence of tri-, di-, and monovalent cations, in this order, could increase the intensity of the acquired excitation and/or emission signals. This behavior becomes of interest if we consider how the use of such an agent, for the detection of the pH, should be applied for both an intra- and extra-cellular environment, in which a considerable difference in the amount of the discussed cations is present.

It is well known that Na^+^ is one order of magnitude different in the intra- and extra-cellular compartment (ca. 14 vs. 140 mM intra vs. extra) [50]. Therefore, the use of HPTS in the extracellular compartments requires caution for the strong effect of the high amount of Na^+^ in this compartment. On the contrary, when present in the intracellular space, the low amount of Na^+^ does not influence HPTS fluorescence.

Analogously, the cytoplasmic Ca^2+^ is ca. 100 nM, which is 10^4^ times lower than in the extracellular space (ca. 1.2 mM) [51]. The intracellular concentration can increase up to 1 μM or more under the influence of various cell stimuli such as membrane depolarization, extracellular signaling molecules, or intracellular messengers [51].

For these reasons, the use of HPTS as a cell pH sensor requires the right calibration curves carried out in the proper medium that considers the specific presence of such cations.

The strong dependence of HPTS fluorescence from trivalent cations suggested developing a method for the quantitative assessment of Gd^3+^ ions present in solution or for the qualitative assessment of the same ions present in the intracellular compartments.

Gd^3+^ is a paramagnetic metal widely used to prepare contrast agents for MRI (Gadolinium-based MRI contrast agents (GBCAs)) [23,26]. Since it is toxic when present in free form, lab good practice rules establish that its concentration as free metal must be < 0.3% mol/mol in the preparation of a pharmaceutical formulation employed for application in cellular systems and animals. In this way, the development of a method is needed to guide the synthesis of Gd complexes and to assess the eventual presence of free Gd^3+^. Some years ago, a spectrophotometric method based on xylenol orange was reported as able to quantify the presence of free Gd^3+^ by variation of xylenol orange color from yellow to violet [48].

The herein reported fluorescence method based on HPTS can work in an analogue way by sensing free Gd^3+^ ions with an LoD that is two-fold lower than the one occurring by using xylenol orange.

Strictly closed to the above reported application, there is the possibility to use this method for the quantification of Gd^3+^ freely present in the environment as a pollutant (in water and food) [52]. GBCAs used for MRI are excreted by the body though the renal route and eliminated into urine. Hence, they pass into the sewer system, but they are not blocked by wastewater-treatment systems and emitted into the aquatic ecosystem, with a large influence on fauna and flora [52]. In big cities, close to hospitals where Gd-contrasted MRI are largely carried out, the occurrence of large amounts of Gd was assessed in surface waters (up to 1100 ng/L), sediments (up to 90.5 μg/g), and living organisms. This requires caution and the development of method, such as the one herein reported, for the quantification of free Gd^3+^ ions.

Finally, it is important to evaluate Gd^3+^ intracellularly distributed and eventually released by Gd-based linear and macrocyclic contrast agents. It has been previously reported how Gd-complexes can be degraded when intracellularly entrapped, especially inside macrophages that own a complex enzymatic armory able to attack the xenobiotic molecules [28]. Previously, our group showed that a different fate is present for Magnevist^®^, Omniscan^®^, and ProHance^®^ entrapped inside macrophages, where the less stable neutral linear Omniscan^®^ is rapidly degraded immediately upon internalization. The more stable linear and negatively charged Magnevist^®^ is degraded at a lower extent and only at longer times upon entrapment. Conversely, the neutral macrocyclic ProHance^®^ was shown to be stable for very long times.

Herein, we tested the HPTS capability to sense free Gd^3+^ ions eventually present inside murine macrophages J774A.1 upon labeling with the three Gd complexes above reported. Cells were cultured under five different conditions: medium only, medium added with HPTS, and added with HPTS plus each of the three contrast agents Magnevist^®^, Omniscan^®^, and ProHance^®^. The fluorescent dye HPTS was eventually placed in medium at the concentration of 50 µM and Gd complexes at 30 mM. We found an analogue number of internalized HPTS for all specimens, corresponding to 3–4 × 10^7^ HPTS/cell. On the contrary, a slight difference in the number of internalized Gd complexes for cells was found, with a slightly higher amount upon incubation in the presence of ProHance^®^ (5.3 × 10^10^ Gd^3+^/cell) with respect to the other two complexes (4.4 × 10^10^ Gd^3+^/cell).

As reported in Figure 8, only in specimens incubated in the presence of the less stable Omniscan^®^ is there an enhancement of HPTS fluorescence. This finding agrees with the release of Gd^3+^ by Omniscan^®^ that we expect after five hours, which is something negligible in all the other cases. It is worth noting that the effect of endogenous Na^+^ and Ca^2+^ on masking Gd^3+^ effect is herein negligible, because intracellularly, these cations have a concentration which is significantly lower than the one reported to be able to mask the effect of Gd^3+^ on HPTS fluorescence (i.e., >20–30 mM for Na^+^ and > 5 Ca^2+^, higher than the intracellular amounts).

To confirm this issue, when cells were lysed and intracellular content was diluted into the external PBS buffer, containing ca. 140 mM Na^+^, the HPTS is not able to sense Gd^3+^ ions, and the fluorescence spectra are almost overlapped for all specimens incubated with the different Gd complexes and HPTS or only HPTS (see Supplementary information).

Altogether, the reported data show how caution is required when using HPTS as a pH reporter and how this fluorescence dye can be usefully employed as a cation sensor in different conditions, opening the avenue to innovative applications of such a probe and the development of new analytical tools.

## 4. Materials and Methods

### 4.1. Chemicals and Buffers

8-Hydroxypyrene-1,3,6-trisulfonate (HPTS), sodium chloride (NaCl), calcium chloride (CaCl_2_), gadolinium chloride (GdCl_3_), lanthanum chloride (LaCl_3_), potassium chloride (KCl), N-(2-Hydroxyethyl) piperazine-N′-(2-ethanesulfonic acid) (Hepes), dipotassium hydrogen phosphate (K_2_HPO_4_), potassium dihydrogen phosphate (KH_2_PO_4_), bovine serum albumin (BSA) and all other chemicals were purchased from Sigma Aldrich. Dulbecco’s Modified Eagle’s medium (DMEM), fetal bovine serum (FBS), penicillin/streptomycin mixture (pen/strep), glutamine (Gln) and MycoAlert™ Mycoplasma Detection Kit were purchased from Lonza Sales AG-EuroClone S.p.A., Milano, It.

Hepes buffer contains Hepes 3.8 mM and NaCl 150 mM. pH was 7.3 ± 0.1, and osmolarity was 280 ± 20 mOsm/L).

PBS buffer contains NaCl 0.137 mM, KCl 2.7 mM, K2HPO4 10 mM, and KH_2_PO_4_ 1.8 mM. pH was 7.3 ± 0.1, and osmolarity was 280 ± 20 mOsm/L.

Hepes without NaCl contains Hepes 3.8 mM in water. pH was 6.5 ± 0.1, osmolarity was 22 ± 20 mOsm/L.

pH was checked by using three-point calibration with standard solutions (pH = 4.0, 7.0 and 10.0).

Osmolarity was checked by using a Knauer K-7400S Semi-Micro Osmometer upon three-point calibration with standard solutions (0, 300, 850 mOsm/L).

For cellular experiments, DMEM was supplemented with a 10% FBS heat-inactivated, penicillin/streptomycin (100 U/mL penicillin and 100 mg/mL streptomycin), and glutamine (2 mM). Cells were negative for mycoplasma as tested by using MycoAlert™ Mycoplasma Detection Kit (Lonza Sales AG-EuroClone S.p.A., Milano, It).

MRI contrast agent used were Magnevist^®^ (Bayer, 469 mg/mL), Omniscan^®^ (GE Healthcare, 287 mg/mL) and ProHance^®^ (Bracco, 279.3 mg/mL).

### 4.2. Fluorescence Measurements

Fluorescence excitation and emission spectra have been acquired by using a Horiba Jobin Yvon Fluorometer. Excitation spectra have been acquired in the 350–495 nm range, with a λ^em^ = 511 nm and a slit width of 1 or 2 nm. Emission spectra have been acquired in the 470–600 nm range, with a λ^exc^ = 422 or 450 nm and a slit width of 1 or 2 nm.

8-Hydroxypyrene-1,3,6-trisulfonate (HPTS) was dissolved in different buffer solutions at the final concentration of 100 nM and placed into plastic four clear sides cuvettes.

For titration in the presence of cations, NaCl, CaCl_2_, GdCl_3_, or LaCl_3_ were added to the HPTS water solution, and pH was maintained at 6.0 to avoid metal hydroxide precipitation.

Blank specimens (i.e., HPTS in water w/o additional cations) were tested 10 times to establish the mean value of blank ± 3 σ.

### 4.3. Linearization of Data

The distribution of the points in the scatterplot suggests that the function describing the behavior of the points is not linear, but that can be of the exponential type.

When the experimental data do not show a linear behavior, the extrapolation of the parameters could be at least difficult, and in the chemical field, the data acquisition represent the first step of the analysis.

It seems to be convenient in these terms to do a conversion of the equation of the curve in a straight line doing a change of variables, a linearization.

Considering the function

y = Ae^Bx^ (2)

We can apply a linearization of the function applying the logarithmic function

lny = lnA + Bx(3)

Placing

Y = lny, C = lnA, X = x(4)

We can graph the new equation

Y = C + BX(5)

For our data, following the same principle the linear equation will be

y = y0 + Ae^R0x^ (6)

y0 − y = −Ae^R0x^ (7)

ln(y0 − y) = ln(−A) + R0x(8)

Y = A + Bx(9)

Y = ln(y − y0)(10)

A = ln(−A)(11)

B = R0(12)

Macrophage cell line J774A.1 was considered for cellular experiments, and cells were obtained from the American Type Culture Collection (ATCC, Manassass, VA, USA).

Cells were grown in enriched DMEM and seeded in 75 cm^2^ flasks at a density of 14 × 10^4^ cells/cm^2^ in a humidified 5% CO_2_ incubator at 37 °C. Once the confluence was reached, cells were detached by using a scraper and split in flasks to obtain a sufficient number of replicates to work with.

### 4.4. Loading of Gd-Based Agents and HPTS Fluorescent Dye

About 5 × 10^5^ cells were seeded on 10 cm Petri dishes, and after 2 days, they were placed in five different incubation conditions (Figure 7) in a humified 5% CO_2_ incubator at 37 °C for five hours:

- Only medium to be used as a control (three replicates);
- Medium enriched only with HPTS 50 µM (four replicates);
- Medium enriched with HPTS 50 µM and Magnevist^®^ 30 mM (four replicates);
- Medium enriched with HPTS 50 µM and Omniscan^®^ 30 mM (three replicates);
- Medium enriched with HPTS 50 µM and ProHance^®^ 30 mM (three replicates).

After the incubation, cells were extensively washed with fresh PBS for the removal of the non-internalized fraction of agents and fluorescent dyes, mechanically detached by scraper, suspended in 3 mL of PBS, and used for pre- and post-lysis acquisitions.

### 4.5. Lyophilization and Cell Lysis

After the spectra acquisitions, cell lysates have been lyophilized by Heto LyoLab 3000 vacuum centrifuge and samples suspended in 200 µL of double distilled water. Then, they were lysed by sonication using a Bandelin Sonoplus probe sonicator (20 KHz) for 20 s at 30% of the power in ice. The cell lysates of each specimen were used for quantification of the cells number (by Bradford Assay) and intracellular content of Gadolinium.

### 4.6. Bradford Assay for Cells Quantification

The number of cells in the specimens was assessed by quantifying the amount of proteins by Bradford Assay.

Absorbance measurements related to the Bradford assay were carried out on a 6715 UV/Vis Spectrophotometer JENWAY. Acquisitions were at 595 nm in plastic two-face cuvettes. After cell lysis on the samples with sonication, the Bradford assay allowed the quantification of cell number. For the baseline acquisition, a solution made by 1 mL of BIO-RAD Protein Assay Dye Reagent and 20 µL of NaCl 0.15 M was considered. All the cell samples were prepared with 1 mL of the same BIO-RAD reagent added to 20 µL of each lysate. Each measurement was performed in replicate. Bovine serum albumin solutions were used as standard. A calibration curve was previously recorded for obtaining the number of cells from the total amount of protein. For J774A.1 cells, 1 mg of protein is equal to 2.5 × 10^6^ cells.

### 4.7. Quantification of the Intracellular Gadolinium

Cells lysates were treated with HCl 37% (50:50 *v*:*v*) in sealed vials at 120 °C overnight to cause destruction of the MRI agents complexes and the release of free Gd^3+^ aqua-ions (mineralization).

The *R*_1_ of the solutions was measured at 21 MHz, 25 °C, with a Stelar Spin Master Relaxometer (Mede, Pavia, Italy). Gd^3+^ were calculated by using calibration curves obtained from standard solutions of GdCl_3_ in chloride acid.

### 4.8. Software and Tools

OriginPro (software version 9.85) was statistical and graphical tool of reference.

## 5. Conclusions

In conclusion, in this work, the influence of media and in particular cations on HPTS fluorescence has been tested. This is important for the use of this probe in the determination of cellular pH in order to avoid misleading results.

It has been demonstrated that an important role in HPTS fluorescence is due to the presence of cations in the monovalent > divalent > trivalent order.

Particularly, in the presence of free Lanthanide ions, there is a strong enhancement of fluorescence. Therefore, we have demonstrated the feasibility of using HPTS for revealing the presence of free gadolinium ions in solution. This can be employed in the chemical synthesis of GBCAs for MRI as a tool to test the eventual presence of unchelated Gd(III) ions and for the assessment of intracellular degradation of GBCAs.

This opens the avenue to a new application of HPTS for the quantification of cations in biological specimens.

## Figures and Tables

**Table 1:** Extracted pKa values for HPTS in different media.

Solvent	pKa
H_2_O	7.85
HEPES	7.84
BSA	7.75
NaCl	7.15
PBS	7.15
HEPES/NaCl	6.98

## Supplementary Materials

The following supporting information can be downloaded at: https://www.mdpi.com/article/10.3390/molecules27082490/s1, Table S1: Parameters of the sigmoidal fitting for pH variation in different media, Figure S1: Exponential fit on variable HPTS concentration, Figure S2: Focus on the range 1 µM to 20 µM, Table S2: Parameters of the exponential fitting, Table S3: Parameters of the exponential fittings in Figure 2A, Table S4: Parameters of the exponential fitting in Figure 3B, Table S5: Parameters of the exponential fitting in Figure 3C, Table S6: Parameters of the exponential fitting to determine HPTS concentration in solution of the sample, Figure S3: (**A**) Comparison of emission spectra for 0.1 mM of cations and 10 µM HPTS (pH = 6.2 ± 0.1), (**B**) Enhancement of fluorescence emission at λ = 511 nm upon excitation at λ = 450 nm, Figure S4: Calibration curve and exponential fitting for the quantification of the concentration of HPTS in the samples, Figure S5: Spectra acquired after cell lysis, Table S7: Calculated HPTS concentration and the per cell content, Table S8: Calculated total Gadolinium concentration and the *per* cell content, Table S9: Smiles code of the molecules.

## Sample Availability

Samples of all the compounds employed in this work are available from the authors.
