# Supplementary material for: Effects of Cations on HPTS Fluorescence and Quantification of Free Gadolinium Ions in Solution; Assessment of Intracellular Release of Gd3+ from Gd-Based MRI Contrast Agents
Source: Molecules. 2022 Apr 12;27(8):2490. doi: 10.3390/molecules27082490 (PMC9032885; doi:10.3390/molecules27082490)
Supplement: Supplementary file 1 [file molecules-27-02490-s001.zip › molecules-1660503-supplementary.pdf]

molecules

Article

Effects of Cations on HPTS Fluorescence and Quantification of Free Gadolinium Ions in Solution; Assessment of Intracellular Release of Gd3+ from Gd-Based MRI Contrast Agents

Angelo Scarciglia, Enza Di Gregorio *, Silvio Aime and Giuseppe Ferrauto

Molecular Imaging Center, Department of Molecular Biotechnologies and Health Sciences, University of Torino (IT), Via Nizza 52, 10126 Turin, Italy; angelo.scarciglia@edu.unito.it (A.S.); silvio.aime@unito.it (S.A.); giuseppe.ferrauto@unito.it (G.F.)

* Correspondence: enza.digregorio@unito.it; Tel.: +39-0116708459

Table S1. Parameters of the sigmoidal fitting for pH variation in different media

		Value	Standard Error
**HEPES**	A1	$1.49x10^5$	$2.23x10^6$
	A2	$7.87x10^7$	$5.07x10^5$
	x0	7.04	$0.89x10^{-2}$
	dx	0.44	$0.73x10^{-2}$
R^2		0.99852	
**H_2O**	A1	$1.56x10^5$	$8.92x10^5$
	A2	$6.61x10^7$	$1.96x10^6$
	x0	7.87	$0.39x10^{-1}$
	dx	0.40	0.04
R^2		0.99701	
**BSA**	A1	$-1.48x10^{-5}$	$4.76x10^4$
	A2	$5.89x10^7$	$8.65x10^5$
	x0	7.75	0.03
	dx	0.55	0.02
R^2		0.99907	

		Value	Standard Error
**HEPES/NaCl**	A1	$-2.89x10^{5}$	$4.76x10^{5}$
	A2	$7.41x10^{7}$	$8.65x10^{5}$
	x0	7.78	$0.27x10^{-1}$
	dx	0.42	0.02
R^2		0.99981	
**NaCl**	A1	$-1.24971x10^{6}$	$2.89x10^{6}$
	A2	$7.38x10^{7}$	$3.33x10^{6}$
	x0	7.15	0.08
	dx	0.57	0.08
R^2		0.98655	
**PBS**	A1	$3.65x10^{6}$	$1.58x10^{6}$
	A2	$7.21x10^{7}$	$3.48x10^{5}$
	x0	7.15	$0.24x10^{-1}$
	dx	0.44	$1.46x10^{-2}$
R^2		0.99957	
**EQUATION**		$y=(A2 + (A1-A2)) / (1+ e^{((x-x0)/dx)})$	

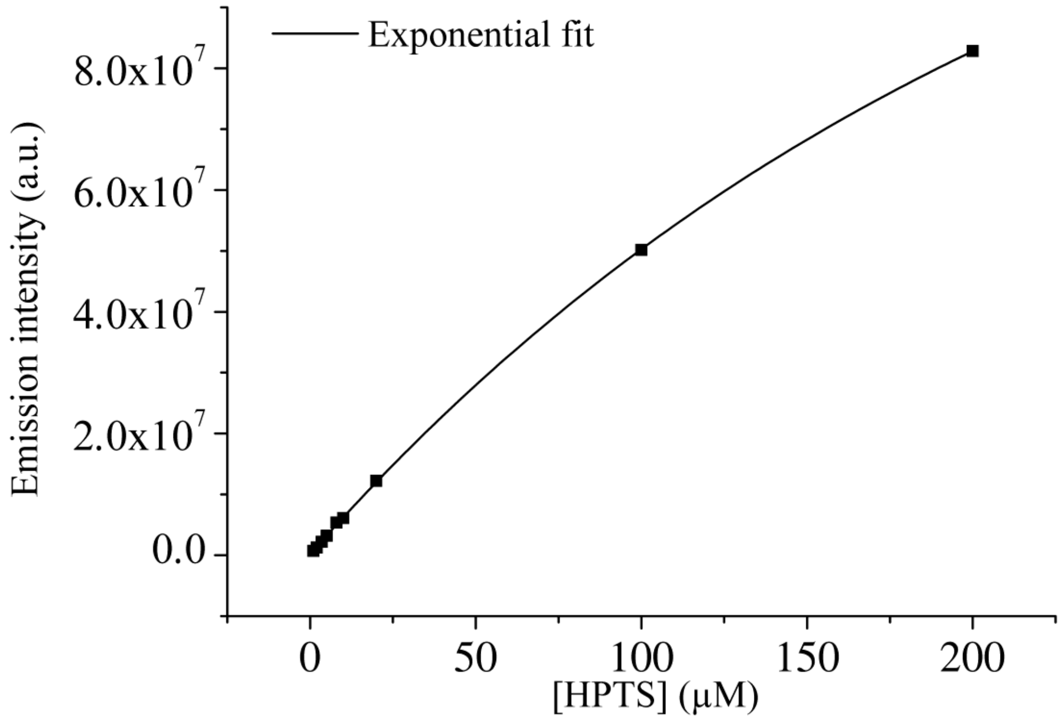

Figure S1. Exponential fit on variable HPTS concentration.

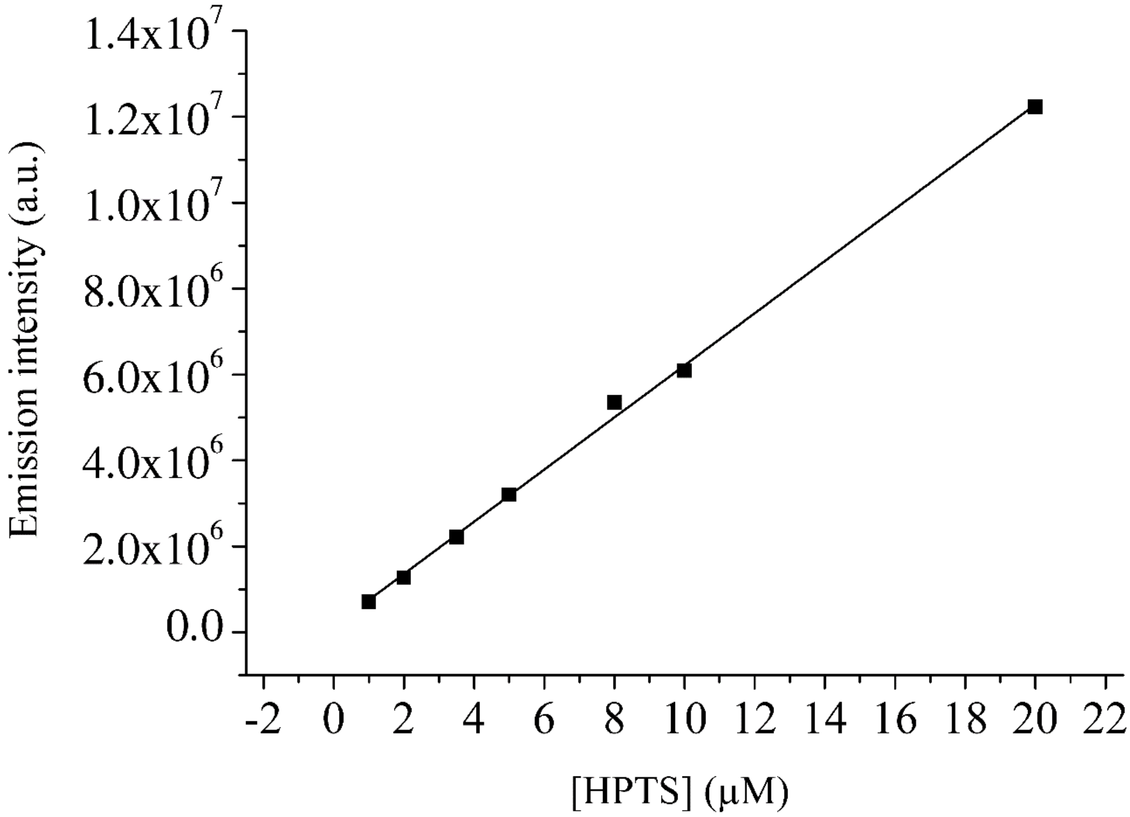

Figure S2. Focus on the range 1μM to 20μM.

Table S2. Parameters of the exponential fitting.

	**yo**	**A**	**R0**	**Reduced Chi-Sqr**	**Adj. R-Square**
**Value**	$1.43x10^{8}$	$-1.43x10^{8}$	$-4.32x10^{-3}$	$3.66x10^{10}$	0.99996
**Standard error**	$2.49x10^{6}$	$2.45x10^{6}$	$1.12x10^{-4}$		
**Equation**			$Y = y0 + Ae^{R0x}$		

Table S3. Parameters of the exponential fittings in Fig. 2A.

		**Value**	**Standard Error**
**Na^{+}**	y0	$1.45x10^{7}$	$1.19x10^{6}$
	A	$-1.15x10^{7}$	$1.13x10^{6}$
	R0	-6.62298	$2.33x10^{11}$
R^2		0.98248	
**Ca^{2+}**	y0	$2.48x10^{7}$	$1.93x10^{6}$
	A	$-2.15x10^{7}$	$2.05x10^{6}$
	R0	-9.5933	2.3314
R^2		0.96894	
**La^{3+}**	y0	$2.41x10^{7}$	$3.29x10^{5}$
	A	$-2.14x10^{7}$	$6.41x10^{5}$
	R0	-30.324	2.748
R^2		0.9956	
**EQUATION**		$y= y_0 + A*e^{(R0*x)}$	

Table S4. Parameters of the exponential fitting in Fig. 3B.

	yo	A	R0	Reduced Chi-Sqr	Adj. R-Square
**Value**	1.94×10^{7}	-1.55×10^{7}	-0.00538	2.45×10^{10}	0.99886
**Standard error**	252784.97	2.36×10^{5}	2.03×10^{-4}		

Table S5. Parameters of the linear fitting in Fig. 3C.

	Intercept	Slope	Adj. R-Square
**Value**	16.5678	-0.0055	0.99905
**Standard error**	0.01027	5.67×10^{-5}	

able S6. Parameters of the exponential fitting to determine HPTS concentration in solution of the sample.

	yo	A	R0	Reduced Chi-Sqr	Adj. R-Square
**Value**	6.84×10^{7}	-6.83×10^{7}	-0.71	4.64×10^{10}	0.99991
**Standard error**	2.56×10^{5}	2.57×10^{5}	6.45×10^{-3}		
**Equation**			$Y = y0 + Ae^{R0x}$		

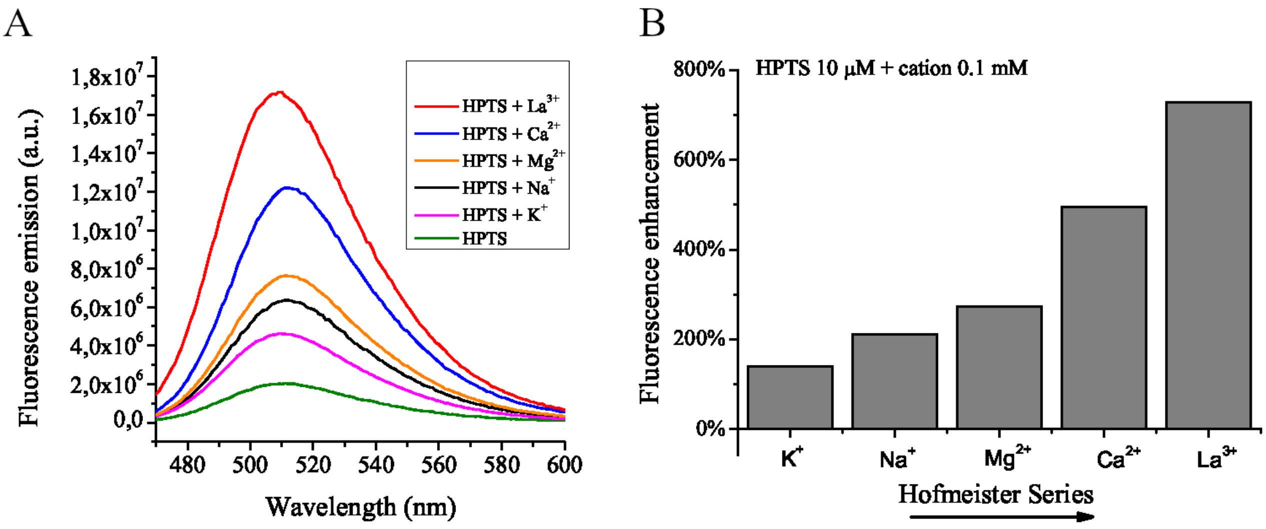

Figure S3. (A) Comparison of emission spectra for 0.1 mM of cations and 10 µM HPTS (pH=6.2±0.1), (B) Enhancement of fluorescence emission at λ=511 nm upon excitation at λ=450 nm.

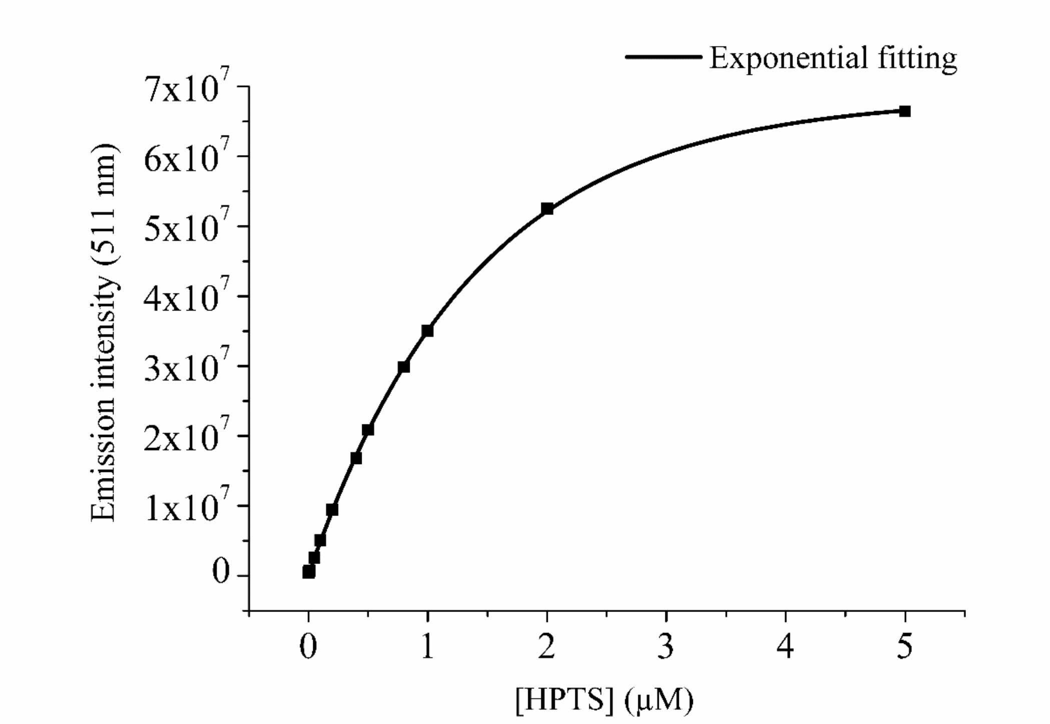

Figure S4. Calibration curve and exponential fitting for the quantification of the concentration of HPTS in the samples

Figure S5. Spectra acquired after cell lysis

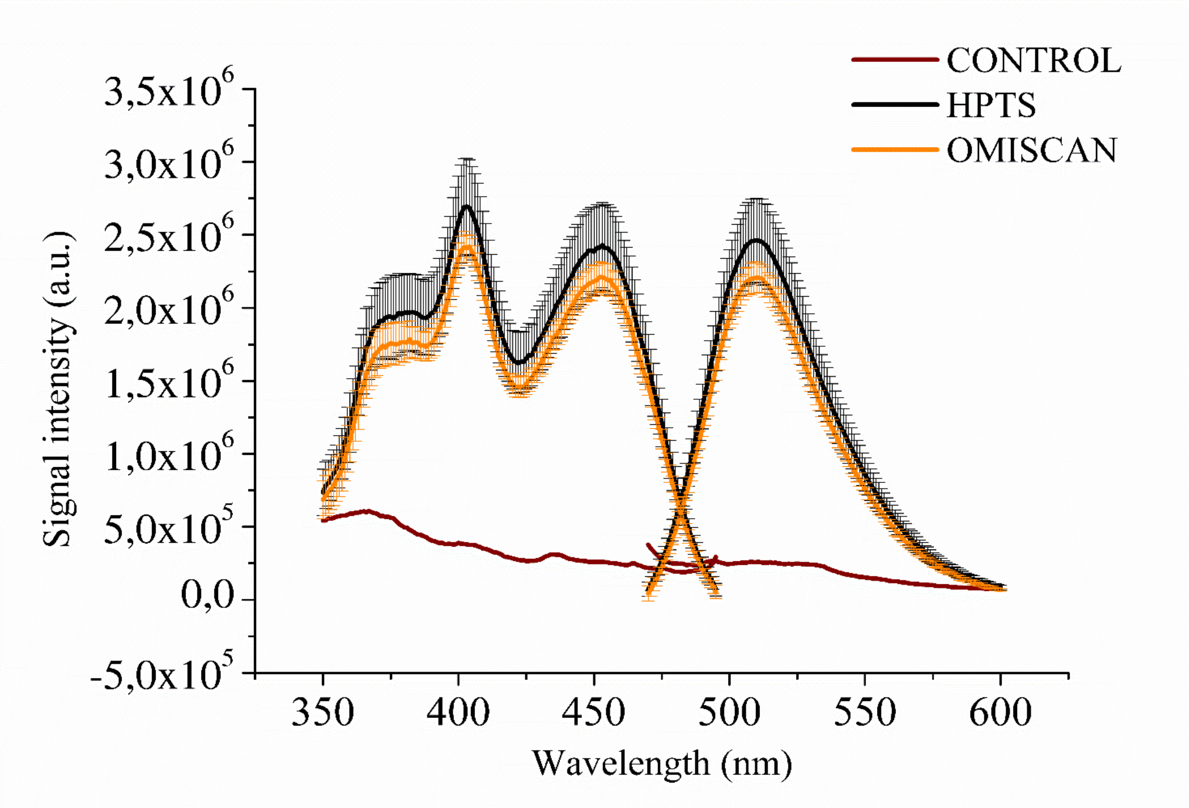

Table S7. Calculated HPTS concentration and the *per* cell content.

SAMPLE	Emission (511 nm)	Cell number	[HPTS] (μM) in 3ml solution	Moles in 3ml solution	Number of HPTS molecules per cell	Moles of HPTS molecules per cell
**WHITE**	$2.59x10^{5}$	$2.20x10^{6}$	0.0018	10^{-12}	-	-
**HPTS**	$2.46x10^{6}$	$1.82x10^{6}$	0.047	$1.41x10^{-10}$	$4.66x10^{7}$	$7.74x10^{-17}$
**MAGNEVIST**	$1.49x10^{6}$	$8.68x10^{5}$	0.027	$8.1x10^{-11}$	$5.61x10^{7}$	$9.33x10^{-17}$
**OMNISCAN**	$2.20x10^{6}$	$1.34x10^{6}$	0.042	$1.26x10^{-10}$	$5.67x10^{7}$	$9.42x10^{-17}$
**PROHANCE**	$1.64x10^{6}$	$1.24x10^{6}$	0.03	$9x10^{-11}$	$4.37x10^{7}$	$7.26x10^{-17}$

Table S8. Calculated total Gadolinium concentration and the *per* cell content.

SAMPLE	Cell number	[Gd] (mM) in 3ml solution	R1	Error	Moles in 3ml solution	Number of Gd atoms/molecules per cell	Moles of Gd atoms/molecules per cell
**HPTS**	$1.82x10^{6}$	0.018	0.62	$1.25x10^{-3}$	-	-	-
**MAGNEVIST**	$8.68x10^{5}$	0.28	2.44	$4.5x10^{-3}$	$5.67x10^{-8}$	$3.93x10^{10}$	$6.53x10^{-14}$
**OMNISCAN**	$1.34x10^{6}$	0.49	3.88	$7.6x10^{-3}$	$9.88x10^{-8}$	$4.45x10^{10}$	$7.39x10^{-14}$
**PROHANCE**	$1.24x10^{6}$	0.54	4.22	$9x10^{-3}$	$1.08x10^{-7}$	$5.27x10^{10}$	$8.76x10^{-14}$

Table S9. SMILES code of the molecules.

MOLECULE	SMILES CODE
**HPTS**	c1cc2c(cc(c3c2c4c1c(cc(c4cc3)S(=O)(=O)[O-])O)S(=O)(=O)[O-].[Na+].[Na+].[Na+]
**MAGNEVIST®**	[Gd+3].OC(=O)CN(CCN(CCN(CC(O)=O)CC([O-])=O)CC([O-])=O)CC([O-])=O
**OMNISCAN®**	[Gd+3].CNC(=O)CN(CCN(CCN(CC([O-])=O)CC(=O)NC)CC([O-])=O)CC([O-])=O
**PROHANCE®**	[Gd+3].CC(O)CN1CCN(CC([O-])=O)CCN(CC([O-])=O)CCN(CC([O-])=O)CC1
